# Window of audio-visual simultaneity is unaffected by spatio-temporal visual clutter

**DOI:** 10.1038/srep05098

**Published:** 2014-05-29

**Authors:** Erik Van der Burg, John Cass, David Alais

**Affiliations:** 1 School of Psychology, University of Sydney, NSW 2006, Australia; 2 School of Social Sciences and Psychology, University of Western Sydney, NSW 2214, Australia

## Abstract

In the present study we investigate the rules governing the perception of audiovisual synchrony within spatio-temporally cluttered visual environments. Participants viewed a ring of 19 discs modulating in luminance while hearing an amplitude modulating tone. Each disc modulated with a unique temporal phase (40 ms intervals), with only one synchronized to the tone. Participants searched for the synchronised disc whose spatial location varied randomly across trials. Square-wave modulation facilitated search: the synchronized disc was frequently chosen, with tight response distributions centred near zero-phase lag. In the sinusoidal condition responses were equally distributed over the 19 discs regardless of phase. To investigate whether subjective synchrony in the square-wave condition was limited by spatial or temporal factors we repeated the experiment with either reduced spatial density (9 discs) or temporal density (80 ms phase intervals). Reduced temporal density greatly facilitated synchrony perception but left the synchrony bandwidth unchanged, while no influence of spatial density was found. We conclude that audio-visual synchrony is not strongly constrained by the spatial or temporal density of the visual display, but by a temporal window within which audio-visual events are perceived as synchronous, with a full bandwidth of ~185 ms.

We receive a continual stream of information in our different sensory modalities, some of which are related to single sources. The brain seeks to combine common crossmodal signals to benefit from the heightened perceptual processing afforded by multisensory interactions^1^,^2^. One indication of a common source is the presence of highly spatially and temporally correlated signals between senses (e.g., as when seeing and hearing a barking dog at a distance of ten meters or less). However, multisensory signals that are only approximately aligned in time may still interact^3^,^4^,^5^,^6^,^7^,^8^,^9^. In fact, perfect temporal alignment is not a prerequisite, as long as the multisensory events are presented within the so-called temporal window of multisensory integration^10^,^11^. This window is useful as many internal and external factors can alter the timing correspondence of multisensory signals (distance, intensity, contrast, adaptation, etc.) rendering perfect crossmodal alignment highly unlikely in natural environments.

The majority of studies looking at multisensory interactions have used single stimuli in each modality (e.g., one sound with one visual event^9^,^10^,^11^,^12^). In real world situations, however, our brains deal with a far richer multisensory environment and the scope for spurious associations is greater (e.g., following a conversation in a room full of speakers). As a result, multiple auditory and visual events may appear within the temporal window of integration, and therefore compete with each other. Recent studies have begun to examine multisensory interactions using more complex audiovisual stimuli containing multiple stimuli^13^,^14^,^15^,^16^,^17^. In one of these studies, the ‘pip and pop' paradigm was introduced as a convenient framework for examining multisensory interactions in cluttered displays^17^. An array of visual elements that flicker repeatedly and asynchronously with respect to one another is paired with a single auditory signal synchronized with one of the visual stimuli. Although this target is visually unique (defined by an orientation, for example), it may be difficult to find in a purely visual search because of the clutter of surrounding elements. However, playing a tone ‘pip' in synchrony with an abrupt temporal change in the target makes it ‘pop' out and the search becomes quick and efficient^17^,^18^,^19^,^20^,^21^,^22^. The essence of this effect is crossmodal binding through audiovisual synchrony^23^, as the auditory signal itself is spatially uninformative about the target's location, and because substituting the auditory cue with a visual cue is not effective^17^,^24^.

In a recent study, Van der Burg et al.^24^ examined whether a single auditory signal can guide attention towards multiple visual events. In that study, a single auditory event was synchronized with one or multiple objects that changed colour (1–8 objects), and participants were subsequently required to identify the locations of the synchronized objects. Interestingly, accuracy was high when a single element was synchronized, but declined dramatically when more than one element was synchronized with the sound. These results indicate that even though multiple synchronized visual matches may be present within the audio-visual synchrony window, only one visual event can integrate with the auditory signal.

Here we adapt the pip and pop paradigm to investigate the spatial and temporal precision of synchrony driven search in cluttered scenes. First, rather than asking participants to respond as quickly as possible to the target's identity (e.g., its orientation), we had participants indicate the location of the target (the one whose luminance modulation is synchronized with the sound). Second, we analyse the spatiotemporal distribution of these responses. Even if audiovisual synchrony is effective at highlighting the flickering visual target, a proportion of errors may still occur such that one of the disks which is not perfectly synchronized with the tone is selected. Indeed, two kinds of errors could arise. In one, attention may be drawn to the target's general spatial vicinity but not necessarily precisely to the target. Consequently spatially neighbouring disks may be selected even though the phase of their temporal modulation may be distant from that of the modulating tone. Alternatively, an error could occur because participants select an element that is nearly synchronous (i.e. whose phase is within close temporal proximity) with the auditory cue, even though it may be spatially distant from the target. The first kind of error would occur if spatial proximity was prioritised, and the second kind would indicate temporal proximity is prioritised and spatial proximity is irrelevant. If the latter is true, then plotting distributions of target location judgments in terms of phase will reveal a tight cluster of synchrony responses located near the point of physical synchrony. The peak of this distribution will indicate the point of subjective synchrony and its bandwidth will reveal the temporal window of subjective synchrony. If spatial proximity contributes to synchrony judgements (i.e., errors of the former kind) then the distribution of perceived synchrony over temporal phase will be much broader.

## Experiment 1

**Figure 1:**
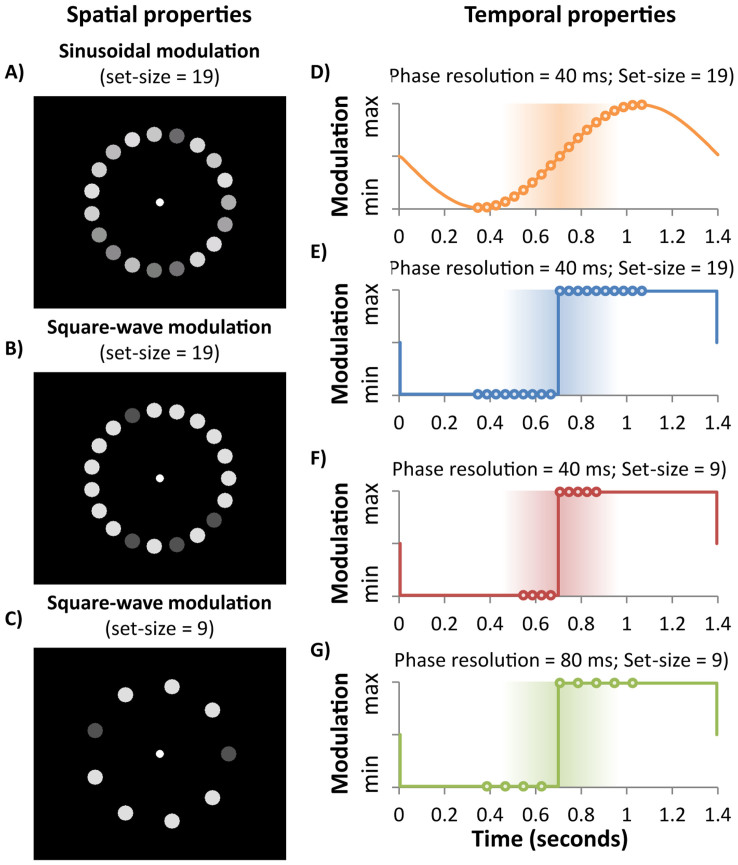
Spatial and temporal properties of the stimulus displays. (A–C) Spatial properties: Displays contained either 19 discs or 9 discs, equally spaced at a radius of 4.4° from fixation. Experiment 1 used displays containing 19 discs and compared square-wave audiovisual modulations with sinusoidal modulations, while Experiment 2a used 9 discs and compared two different densities of temporal change. Experiment 2b compared 9 vs. 19 discs for square-wave audiovisual modulations. (D–G) Temporal modulation properties: The auditory signal and all of the discs modulated at 0.71 Hz. Experiment 1 used 19 discs and therefore the modulation wavelength was divided into 19 equally spaced phase points so that each disc had a unique phase. The auditory signal's phase was set to 0 ms, matching the zero-phase visual disc to create a synchronized audiovisual target. Experiment 2a used 9 discs and compared two different temporal densities sampling phase in either 40 ms or 80 ms intervals around the zero-phase point. Experiment 2b used 9 or 19 discs with a similar temporal density (40 ms intervals). The dots plotted on the modulation waves represent the phases allocated to the discs in the display. Note that the spatial allocation of phases to discs was random, not sequential. The shaded central bands signify a feasible temporal window of integration.

Participants saw 19 discs in the display (see Fig. 1A,B), each modulating with a unique temporal phase shift. Half of the modulation wavelength was divided into 19 equally spaced phase steps (−360–+360 ms in steps of 40 ms) and spatially randomized among the 19 display locations. The auditory modulation always had a phase offset of 0 ms (making the zero-phase disc the visual target). After three cycles of modulation, the discs disappeared and participants identified the location of the disc that appeared synchronized with the tone. The spatial order of the phase shifts was re-randomised every trial, as was the starting point of the audiovisual modulation sequence. The first experiment compares two audiovisual conditions: one in which both auditory and visual modulation components are square-wave (see Fig. 1E) and another in which both are sinusoidal (see Fig. 1D). Previous work suggests that square-wave, but not sine-wave, modulations should produce synchrony-driven audiovisual binding^19^, however this finding was established in an experiment that used displays far less spatially and temporally dense than those used here, and which also used a different dependent variable (reaction time, rather than perceived synchrony as used here). Experiment 1, apart from providing a useful replication of the sine- vs. square-wave difference, will demonstrate the importance of square-wave modulation in auditory-visual binding more directly by actually measuring perceived synchrony rather than reaction times.

### Method

#### Participants

Twelve participants (4 female, mean age 33.1 years; age range 19 to 49 years) participated in the experiment, including nine who were naïve to the experiment's purpose. Ethical approval for this study was obtained from the ethical committee at the University of Sydney. The experiments were conducted according to the principles laid down in the Helsinki Declaration. Written informed consent was obtained from all participants except the authors.

#### Stimuli and apparatus

Participants were seated in a dimly lit room approximately 80 cm from the monitor (75 Hz refresh rate) and wore headphones. Arrays of discs were presented on an imaginary circle of radius 4.4° of visual angle around fixation, with each disc (0.52° of visual angle in radius) modulating in luminance from 10.1–95.4 cd m^−2^ at a rate of 0.71 Hz (see Fig. 1) on a black background (<.5 cd m^−2^). An auditory signal (a 500 Hz tone varying in intensity between silence and 73 dB) modulated at 0.71 Hz and was present in all trials. In Experiment 1 there were 19 discs in the display, and both components of the audiovisual signal were either sinusoidally modulated or square-wave modulated (see Fig. 1D,E). Importantly, each disc modulated with a unique temporal phase. As shown in Figure 1D,E, the modulation wavelength was divided into 19 equally spaced phase steps (−360–+360 ms in steps of 40 ms). The auditory modulation always had a phase offset of 0 ms (making the zero-phase disc the visual target). The spatial location of each phase offset was randomized on every trial, as was the starting point of the audiovisual modulation sequence, such that the ‘zero-phase' may occur at any point in the stimulus cycle and therefore at any location. The experiment was programmed and controlled by E-prime software.

#### Design and procedure

Each trial began with a central fixation dot for 1,000 ms followed by the array of modulating discs and accompanying auditory signal. After three cycles of modulation (4.225 s), the discs disappeared and were replaced by the numbers 1–19 and the task was to identify the location of the disc that was synchronized with the tone by reporting its number. The dependent variable was the proportion of responses for each phase offset. Modulation type (sinusoidal vs. square-wave) was randomly mixed within blocks of 32 trials and 13 experimental blocks were completed (208 trials for each modulation condition). Before the experiment began, participants completed 5 practice blocks of 16 trials and feedback was given after each trial.

### Results

**Figure 2:**
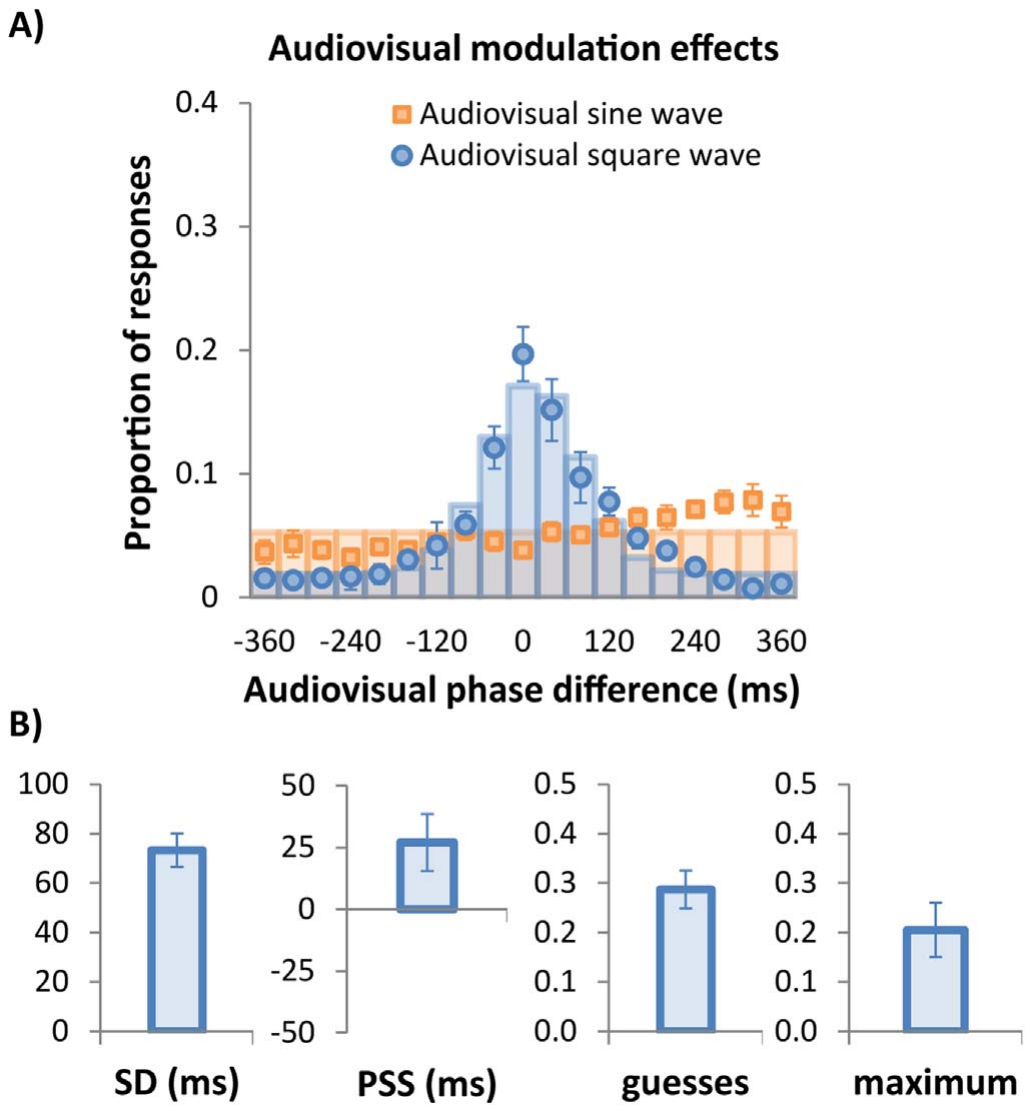
Results of Experiment 1. (A) Data points show group mean (n = 12) proportion of ‘target' responses as a function of the 19 audio-visual phase differences. Negative phase differences indicate the visual modulation preceded the auditory modulation, positive phase differences indicate the visual signal lagged the auditory one. Orange squares show sinusoidal audiovisual modulations, blue symbols indicate square-wave modulations. Blue columns show the best-fitting Gaussian function defined by Equation 1. No Gaussian fit was possible for the sine-wave data: orange columns indicate uniform chance-level performance at 0.053 (i.e., 100% guesses). (B) Group mean parameter estimates after fitting each participant's data in the square-wave condition with the three-parameter Gaussian model shown in Equation 1. Maximum amplitude was not fitted but corresponds to the peak of the best-fitting equation. The guessing rate is calculated by multiplying y0 with the set size. Error bars represent ±1 standard error.

Figure 2A plots the proportion of synchrony responses for each of the 19 visual phase points, for both the sine-wave (orange data points) condition and square-wave (blue data points) condition averaged across subjects. The auditory signal has a phase of 0 ms and negative values indicate the visual modulation was advanced relative to the auditory modulation. An ANOVA on proportion of synchrony responses with modulation type and phase as within-subject variables was conducted. The ANOVA yielded a significant two-way interaction, F(18, 198) = 14.9, p < .001. This interaction was further examined by separate ANOVAs for each modulation type. For the square-wave condition, the ANOVA on phase was highly significant, F(18, 198) = 15.8, p < .0001, indicating that the proportion of responses varied as a function of the phase. For the sine-wave condition, the ANOVA on phase was not significant, F(18, 198) = 2.46, p = .062, indicating that the proportion of responses was equally distributed over the discs regardless the phase of each disc.

In the square-wave condition, each participant's data was sorted by temporal phase and fitted a three-parameter Gaussian distribution (see Equation 1) to estimate the point of subjective simultaneity (PSS: the distribution's mean), the precision of simultaneity judgments (the distribution's standard deviation: SD) and a baseline elevation (y0). Note that y0 reflects the proportion of guesses when multiplied by the set size. 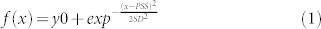
The curve-fitting procedure used Matlab's *fit* function to minimise the sum of squared error and assumed that the total probability under the Gaussian model at the discrete points that were measured summed to 1.0. When the baseline (y0) was elevated above zero, the probabilities under the Gaussian included a uniform baseline component added to each point. Therefore, with overall probability constrained to total 1.0, any baseline elevation necessarily entailed a narrowing of the Gaussian function to offset the rectangular region beneath it. Another consequence of constraining the total probability to 1.0 is that the Gaussian's height will vary inversely with standard deviation. We refer to the height of the best-fitting Gaussian equation as the ‘performance maximum'.

In Figure 2A, the blue columns show the probabilities from the best-fitting Gaussians (see Equation 1) at each of the tested phase points for the group mean data. The unit probability Gaussian model describes the square-wave data very well (r^2^ = 0.951). The Gaussian model was also fitted to the square-wave data of each individual participant and Figure 2B shows the group mean of the precision, performance maximum and simultaneity parameters, together with ±1 standard error bars.

The data in Figure 2A clearly illustrate that square-wave modulation affords far more precise audio-visual synchrony detection than does sine-wave modulation, with the distribution for square-wave modulation being tightly clustered around the PSS (27 ms) with a standard deviation of 73 ms and a guessing rate (i.e., baseline (y0) × 19 elements) of 0.28. These parameters give a performance maximum of 0.21. Confirming an earlier report that transient signals are required for synchrony-driven audiovisual binding to occur^19^, the pattern of data for the sine-wave condition is flat and does not show the expected peak around 0 ms that would indicate synchrony-driven binding. In contrast, the square-wave condition does show a clear peak near 0 ms, indicating that square-wave audiovisual synchrony was effective in guiding attention to the synchronously modulating disc. Together, these results confirm that square-wave modulation affords more accurate audio-visual synchrony detection than does sine-wave modulation.

## Experiment 2

Experiment 1 established the efficacy of square-wave audiovisual modulations for promoting crossmodal binding relative to sinusoidal modulations. However, the task of finding the synchronized disc was still very difficult in the square-wave condition, as evidenced by the fitted maximum of 0.21 being well below 1 (Figure 2A). In Experiment 2 we test whether this somewhat low performance maximum is due to spatial or temporal factors. In Experiment 2A we presented a 9-disc array and manipulated temporal density by comparing 40 ms and 80 ms phase steps to test whether reduced temporal density will improve performance. The modulations in Experiment 1 were tightly spaced in time, separated by 40 ms phase steps, meaning several potential targets could fall within the window of subjective synchrony. This could have restricted the maximum of the synchrony distribution by allowing spurious binding with non-targets that were nearly in phase with the tone. Experiment 2B retains the temporal density used in Experiment 1 but manipulates the display's spatial density by presenting either 9 or 19 discs (see Fig. 1B,C). The discs in Experiment 1 were tightly spaced and peripherally presented, making it difficult to individuate specific discs, and this – rather than temporal density – may have hindered participants' ability to identify the synchronised disc.

## Methods

### Participants

Six participants (2 female, 3 naïve; mean age 34.7 years; ranging from 20 to 47 years) participated in both Experiments 2A and 2B. Informed consent was obtained from all participants.

### Stimuli, apparatus and procedure

The experiments were very similar to Experiment 1 except for the following changes. The sine-wave condition was not tested: all audiovisual modulations were square-wave. In Experiment 2A, the spatial density was fixed by always presenting nine discs but the temporal density was manipulated. The modulating discs were spaced either in 40 ms phase intervals (−160–+160 ms: see Figure 1F) or in 80 ms intervals (−320–+320 ms: see Figure 1G). The two temporal densities were randomly interleaved within blocks. In Experiment 2B, the display's spatial density was manipulated by presenting either 19 discs (spaced and modulated as in Experiment 1) or 9 discs (see Figure 1C). In both spatial density conditions, the modulating discs were spaced in 40 ms phase intervals (as in Experiment 1), however a narrower range of phase steps was used in the nine-disc condition (−160–+160 ms: see Fig. 1F) to keep a constant temporal density within the temporal window of integration (compare Fig. 1E,F). The two set sizes were randomly interleaved within blocks. Participants did a total of 416 trials in each experiment.

## Results

**Figure 3:**
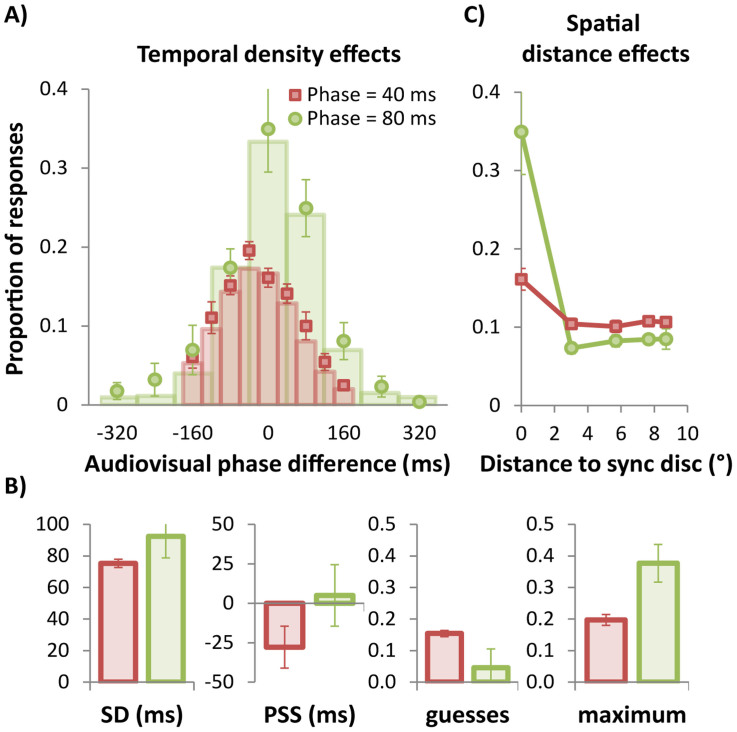
Results of Experiment 2A: Temporal density effects. (A) Mean proportion of ‘target' responses plotted as a function of audio-visual phase difference for displays containing 9 discs. 0 ms indicates the point of physical synchrony. Red symbols show data for phase intervals of 40 ms and green symbols show data for 80 ms intervals, with columns showing the proportions from the best-fitting Gaussian functions (Equation 1) at each of the measured phase points. (B) Group mean parameter estimates after fitting each participant's data with the three-parameter Gaussian model shown Equation 1. Maximum amplitude was not fitted but corresponds to the peak of the best-fitting equation. The guessing rate is calculated by multiplying y0 with the set size. (C) Mean proportion of ‘target' responses in Experiment 2A plotted as a function of spatial distance from the physically synchronised disc. The data show no tendency for the discs spatially adjacent to the synchronised target to be chosen any more than more distant discs. Instead, when errors were made, participants chose a temporally adjacent disc rather than a spatially adjacent disc.

**Figure 4:**
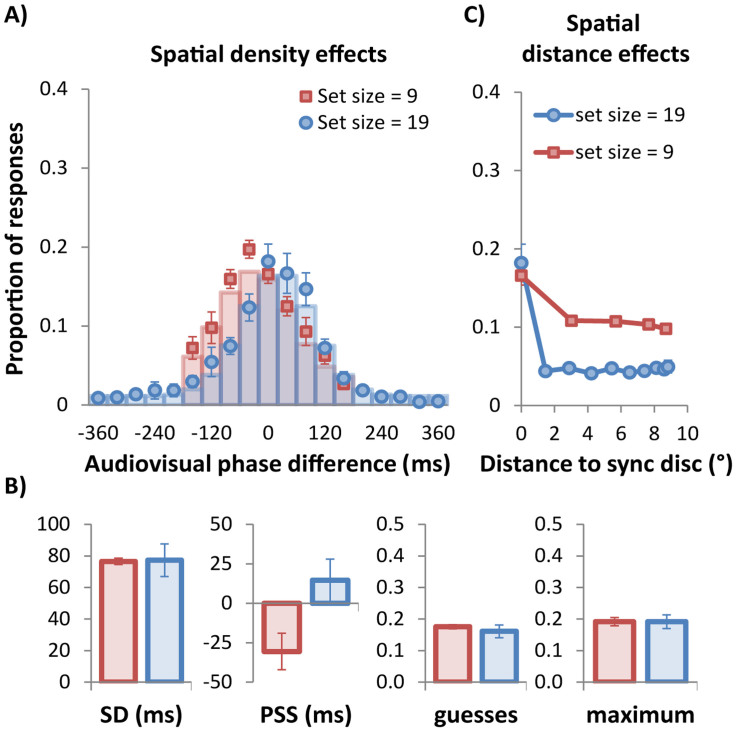
Results of Experiment 2B: Spatial density effects. (A) Mean proportion of ‘target' responses plotted as a function of audio-visual phase difference for displays containing 9 or 19 discs, all modulating with phase intervals of 40 ms. 0 ms indicates the point of physical synchrony. Red symbols show data for a set size of 9 and blue symbols show data for set size of 19, with columns showing the proportions from the best-fitting Gaussian functions (Equation 1) at each of the measured phase points. (B) Group mean parameter estimates after fitting each participant's data with the three-parameter Gaussian model shown Equation 1. Maximum amplitude was not fitted but corresponds to the peak of the best-fitting equation. The guessing rate is calculated by multiplying y0 with the set size. (C) Mean proportion of ‘target' responses in Experiment 2B plotted as a function of spatial distance from the physically synchronised disc. The data show no tendency for the discs spatially adjacent to the synchronised target to be chosen any more than more distant discs.

Data points in Figures 3A and 4A show the results for temporal density manipulation (Experiment 2A) and the spatial density manipulation (Experiment 2B), respectively, with the data points showing group mean proportion of responses as a function of audio-visual phase difference. Columns show the probabilities from the best-fitting unit-probability Gaussians (see Equation 1) at each of the tested phase points for the group mean data. The model describes both distributions in Figure 3A and both in Figure 4A very well, with r^2^ for all conditions > 0.971.

The Gaussian model was also fitted to each individual participant's data. An analysis of the group mean parameters for the temporal density manipulation (Fig. 3B) showed that the temporal spacing (40 ms vs 80 ms) of the modulations did not affect the standard deviation of the best-fitting Gaussian (75 ms vs 92 ms), t(5) = 1.2, p = .267, and neither did it affect the guessing rate (0.15 vs 0.04), t(5) = 1.4, p = .218. There was a trend towards a significant difference between PSS for the two temporal densities (−28 ms vs 5 ms), t(5) = 2.6, p = .050. The strongest effect of the temporal density manipulation was on the performance maximum (0.20 vs 0.38), t(5) = 3.4, p = .019, with the lower temporal density (80 ms phase intervals) producing almost twice the proportion of synchrony responses at the point of subjective synchrony. This shows that temporal density, not spatial density, was the limiting factor in Experiment 1.

We also analysed the spatial distribution of perceived simultaneity judgments. Figure 3C plots the proportion of simultaneity judgments as a function of spatial distance (degrees of visual angle) from the synchronized disc, for both temporal densities. The synchronized disc is indicated by 0° and distance is plotted in absolute terms. An ANOVA on proportion of trials was conducted with distance to synchronized disc (0°, 3.0°, 5.7°, 7.7°, and 8.7°) and phase (40 vs. 80 ms) as within subject variables. The main effect of distance and the interaction were significant, F(4, 20) = 18.0, p = .004 and F(4, 20) = 15.4, p = .004, respectively, indicating that the proportion of responses varied as a function of distance. Importantly, however, when the synchronized disc was excluded from the analysis, neither the main effect of distance nor the two-way interaction were significant, F values < 1. This indicates that the spatial effect was driven solely by the target disc: when participants missed the synchronized disc they had no preference to choose a disc spatially proximate to the synchronized target. Instead, they chose a disc proximate in terms of temporal phase (cf Fig. 3A).

An analysis of the group mean parameters for the Gaussian fits was also conducted for the spatial density manipulation (Fig. 4B) and showed that set size (9 vs 19 discs) did not affect the standard deviation of the best-fitting Gaussian (77 ms vs 77 ms), t(5) = 0.07, p = .947. The spatial density manipulation also did not affect the guessing rate (0.18 vs 0.16), t(5) = 0.1, p = .888 or the performance maximum (.19 vs .19), t(5) = 0.01, p = .991. Spatial density therefore was not a limiting factor in Experiment 1 as the widths of the fitted Gaussians (and therefore the performance maxima) in Experiment 2B did not differ across set sizes of 9 and 19 discs. The only significant difference between set sizes was that the PSS shifted slightly earlier when only 9 discs were present (−31 ms vs 15 ms), t(5) = 4.4, p = .007.

We also conducted an analysis of the spatial distribution of simultaneity judgments for Experiment 2B. Figure 4C plots the proportion of simultaneity judgments as a function of spatial distance from the synchronized disc (i.e., 0°), for both spatial densities (set size = 9 vs. 19). We conducted two separate ANOVAs with distance to the synchronized disc as within subject variable for each set size condition (Set size = 9: 0°, 3.0°, 5.7°, 7.7°, and 8.7°; set size = 19: 0°, 1.5°, 2.9°, 4.2°, 5.4°, 6.5°, 7.4°, 8.1°, 8.6°, and 8.8°). The ANOVA yielded a significant main effect of distance to the synchronized disc in the set size = 9 condition and in the set size = 19 condition, F(4, 20) = 14.2, p < .001, and F(9, 45) = 20.0, p < .001, respectively, indicating the proportion of responses varied with distance from the synchronized disc. Again, the main effect of distance was not significant for either set sizes when the synchronized disc was excluded from the analyses, Fs < 1, indicating that when participants missed the synchronized disc they showed no tendency to choose a disc that was spatially nearby the target.

Note that the data plotted in red in Figure 3 were obtained from identical stimulus conditions to those shown in red in Figure 4 (i.e., set size of 9 and phase interval of 40 ms). As the same participants completed both experiments, we compared these conditions across experiments. Confirming the close agreement evident in the group mean parameter estimates in Figure 3B and 4B (see red columns), there were no significant differences in standard deviation, PSS, baseline or maximum between each data set (two-tailed t-tests, df = 5, all ps > .391).

## Discussion

In the present study we investigated the accuracy and precision with which observers can detect synchronous audio-visual events in spatio-temporally cluttered visual displays. Experiment 1 demonstrated very clearly that audio-visual synchrony detection requires transient signals. This can be seen by the stark difference in Figure 2A between the square- and sine-wave conditions and confirms our earlier result obtained with a similar paradigm but using a less cluttered array and a speeded search task rather than identification of spatial location^19^. The sine-wave data showed no discernible peak as participants' judgements of subjective synchrony were equally distributed over all phases, while the square-wave condition produced a tight cluster of synchrony judgments centered close to the true point of synchrony with a narrow bandwidth of SD = 73 ms. Defining the window of perceived synchrony as the Gaussian function's full-width at half height (see Equation 2) corresponds to a temporal window of 172 ms (that is, 2.35 times the standard deviation), or ±86 ms around the point of subjective synchrony. 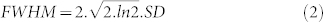
Observers found the task in Experiment 1 extremely difficult and consequently the fitted Gaussian's maximum was quite low at just 0.21. Although this does not affect the bandwidth estimate, as amplitude and bandwidth are independent parameters, we sought to determine whether the window of perceived synchrony could be improved by manipulating the display's spatial or temporal density. Feedback from participants suggested that the closely spaced elements in the 19-item array of Experiment 1 were hard to discern individually, making it difficult to select the synchronized disc accurately. By halving the spatial density from 19 to 9 elements in Experiment 2B, we removed this constraint, yet the distribution of subjective synchrony for 9 elements was remarkably similar to the distribution for 19 elements in both maximum and bandwidth (Fig. 4B). In contrast, Experiment 2A revealed that halving the temporal density from 40 ms to 80 ms between visual events doubled the maximum from 0.20 to 0.38 (Fig. 3B).

Overall, we conducted five square-wave conditions in these experiments, four in Experiment 2 plus the square-wave condition in Experiment 1, and all were remarkably consistent in bandwidth despite large differences in spatial and temporal density. Over the five conditions, the grand mean and standard error for the standard deviation parameter was 79 ms ±3.4 ms. Using this grand mean, we obtain a temporal window from Equation 2 of 185 ms, or ±93 ms around the point of subjective simultaneity. The results of Experiment 2 clearly show that the temporal density rather than the spatial density of visual elements is the limiting factor in task difficulty, as only the temporal conditions produced any change in maxima. However, regardless of whether the stimulus is spatially cluttered or not, or temporally dense or not, the temporal bandwidth of the underlying synchrony mechanism is remarkably constant with an overall bandwidth of ±93 ms. Provided the temporal density is low with respect to this bandwidth, the likelihood of a synchronized audiovisual transient being spatially located will be high^17^,^19^. If the temporal density is high, there will be more than one potential match within the temporal synchrony window and errors will be made. What we have succeeded in demonstrating here is that if an error is made, it is likely to be an element which modulates within close temporal proximity of the synchronized element, regardless of its spatial location.

Previous studies investigating the perceived audiovisual synchrony have typically employed single auditory and visual events^10^,^25^,^26^, although a recent study by Roseboom, Nishida and Arnold^27^ used two visual events in combination with an auditory signal (and *vice versa*). Some other studies have used as many as eight elements^13^,^14^,^28^, however none have investigated multisensory synchrony using stimuli as spatially and temporally dense as those used here. Despite the complexity of our displays, we observe a temporal synchrony window with a full width of about 185 ms (±93 ms), rather similar to the value obtained in studies using relatively simple displays. This suggests that whether there is just one pair of stimuli or many potential pairs competing for synchrony does not affect the window of perceived synchrony, a conclusion supported by our finding in Experiment 2B that temporal integration bandwidth did not differ for arrays of 9 or 19 visual elements. However, temporal density had a profound effect on synchrony detection, as the physically synchronized disc was more often selected as the synchronized element when the temporal density was reduced by a factor two, which we interpret as being due to reduced competition within the otherwise invariant temporal window of perceived synchrony.

In Experiment 2, the estimated PSS was dependent on both temporal and spatial density. This result is somewhat puzzling, but insofar as it is an effect of the number of visual events within the temporal window of perceived synchrony the results are consistent with a study by Roseboom, Nishida and Arnold^27^ who found that perceived simultaneity is contingent upon other visual events within the temporal window of perceived synchrony. Their study examined whether the perceived synchrony of an auditory and standard visual event was affected by the presence of a preceding or lagging (±100 ms) additional visual event within the window of simultaneity. They reported that perceived synchrony between the auditory and standard visual event was shifted away from the additional visual event, making it more likely the auditory signal would go together with the standard visual event than with the additional visual event. This kind of flexibility in the temporal location of perceived synchrony, similar to the PSS shifts shown in Figure 4B, can thus help perceptual segregation across time.

Many audiovisual studies have shown that the temporal window of integration varies among participants^29^,^30^. In Experiment 1, the group mean temporal window with a full width was 171 ms, although individual values ranged from 64 to 251 ms. Apart from confirming that these individual differences are typical, it provides an explanation of individual variation seen in the pip and pop effect (see^31^ for a related discussion). In the pip and pop task, the modulation in the target element is always temporally segregated from the modulating distractors to minimize competition within the window of integration. Therefore, participants with a narrow window of perceptual simultaneity should find the synchronized target more easily as no distractor events would occur inside their window of perceptual simultaneity, whereas participants with a broad window would inevitably experience distractor changes occurring within their window of integration, leading to competition with the target. This also explains why some other studies have failed to find a pip and pop effect as they did not control for temporal overlap between target and distractor modulations^16^,^28^. In these studies, the modulating target and distractors often changed in close temporal proximity, leading to competition and spurious binding within the window of perceived synchrony.

## Author Contributions

E.V.d.B. and J.C. designed research; E.V.d.B. performed research; E.V.d.B. analyzed data; E.V.d.B., D.A. and J.C. wrote the paper. All authors reviewed the manuscript.
